# Enhanced physical health screening for people with severe mental illness in Hong Kong: results from a one-year prospective case series study

**DOI:** 10.1186/1471-244X-14-57

**Published:** 2014-02-27

**Authors:** Daniel Bressington, Jolene Mui, Sabina Hulbert, Eric Cheung, Stephen Bradford, Richard Gray

**Affiliations:** 1 The Department of Health, Well-being and Family, Canterbury Christ Church University, North Holmes Road, Canterbury, Kent CT1 1QU, UK; 2 Nurse Consultant (Community Psychiatric Service), Castle Peak Hospital, 15 Tsing Chung Koon Road, Tuen Mun, New Territories, Hong Kong, China; 3 Research and Enterprise Development Office, Canterbury Christ Church University, North Holmes Road, Canterbury, Kent CT1 1QU, UK; 4 Hospital Chief Executive, Castle Peak Hospital, 15 Tsing Chung Koon Road, Tuen Mun, New Territories, Hong Kong, China; 5 Canterbury Christ Church University, North Holmes Road, Canterbury, Kent CT1 1QU, UK; 6 Department of Nursing Education and Research, P.O. Box 3050 Doha, Qatar

**Keywords:** Physical health screening, Severe mental illness, Health behaviours, Cardiovascular risk

## Abstract

**Background:**

People with severe mental illness have significantly poorer physical health compared to the general population; previous health screening studies conducted outside Asian countries have demonstrated the potential in addressing this issue. This case series aimed to explore the effects and utility of integrating an enhanced physical health screening programme for community dwelling patients with severe mental illness into routine clinical practice in Hong Kong.

**Method:**

This study utilises a consecutive prospective case series design. The serious mental illness Health Improvement Profile (HIP) was used as a screening tool at baseline and repeated at 12 months follow-up.

**Results:**

A total of 148 community-based patients with severe mental illness completed the study. At one year follow-up analysis showed a significant improvement in self-reported levels of exercise and a reduction in the numbers of patients prescribed medications for diabetes However, mean waist circumference increased at follow-up. In addition to the statistically significant results some general trends were observed, including: a lack of deterioration in most areas of cardiovascular risk; a reduction in medicines prescribed for physical health problems; and general improvements in health behaviours over the 12 month period.

**Conclusions:**

The findings demonstrate that using the HIP is feasible and acceptable in Hong Kong. The results of the enhanced physical health-screening programme are promising, but require further testing using a randomised controlled trial design in order to more confidently attribute the improvements in well-being and health behaviours to the HIP.

**Trial registration:**

Clinical trial registration number: ISRCTN12582470

## Background

The poor physical health state of people with severe mental illness (SMI) is well established [1]; people with SMI are at significantly increased risk from a variety of long term physical health problems in comparison to the general population. The increased morbidity of physical health conditions such as cardiovascular disease, obesity, diabetes, metabolic syndrome and hypertension have led to high mortality rates in people with SMI; in schizophrenia, standardised mortality ratios are increased up to four-fold compared with the general population [2-5]. It is estimated that people with SMI have a life-span of between 10 and 30 years less than the general population; which has been observed to be deteriorating over the last decade [6].

In response to the physical health inequalities observed in people with SMI many health care providers in a variety of different countries have produced policies and clinical guidelines which recommend regular physical health screening in this patient group [4,7-9]. The increased focus on the physical health of people with SMI has also resulted in some countries using financial incentives to improve the regularity of screening; for example, in the UK general medical practitioners in primary care settings are offered additional payment for recording the health status of people with SMI [10].

Service improvement and implementation studies have demonstrated that clinicians in mental health services are able to successfully conduct enhanced physical health screening when they are provided with sufficient guidance, training and resources; for example Mangurian et al., [11] reported that 50% of 15,000 outpatients in New York State were able to have Body Mass Index, blood pressure and smoking status recorded over just a four month period. Similarly in the UK; Barnes et al., [12] reported that a quality improvement programme effectively increased levels of screening for metabolic syndrome in community psychiatric patients prescribed antipsychotics. An earlier study conducted in the US [13] reinforces the potential benefits of screening as the results demonstrated that structured health monitoring was effective in detecting physical health problems in people with mental illness.

Once physical health co-morbidities have been identified, interventions to address the problems need to be tailored for patients based on their individual circumstances. Reasons for the poor physical health state of individuals with SMI are multi-factorial; but are likely to relate to a combination of lifestyle issues, diagnostic over-shadowing, adverse effects of prescribed medication, difficulty detecting physical health concerns, patient reluctance to report health problems and the potential impact of psychiatric symptoms on health behaviours [6,14]. A recently published systematic review of the physical health intervention literature [15] concluded that mental health nurses were well-placed to address health behaviours in mental health patients and that specific interventions can result in significant improvements in both health behaviours and physical health outcomes. Although there is currently a lack of evidence from randomised controlled trials [16] the results from less methodologically robust studies suggest that “well-being” initiatives may result in improved patient outcomes [17,18].

Given the close relationship between lifestyle and health, some studies have investigated the use of enhanced screening procedures which combine the identification of both physical co-morbidity and the health behaviours of patients in order to target clinical interventions. With this intention, White et al., [19] developed the serious mental illness Health Improvement Profile (HIP) screening tool and through a pilot study demonstrated the face validity and acceptability of the HIP to both clinicians and patients. The HIP is intended to be used by mental health nurses as a pragmatic physical health risk assessment which aims to direct individualised clinical interventions based on the patient’s physical health state and their responses to the questions about health behaviours. The HIP consists of 27 items which are flagged as “red” for unhealthy (indicating that intervention is required) or “green” for healthy (where no intervention is required).

Subsequently, a pilot study which aimed to establish the clinical utility of the HIP was conducted by Shuel et al., [20] in the UK. The tool was completed with 31 community mental health patients and the results showed that mental health nurses effectively detected physical comorbidity and were also able to use the findings to plan and recommend tailored evidence-based interventions. The study’s qualitative data further demonstrated that using the HIP was acceptable to patients and their clinicians.

The previous physical health screening studies are primarily confined to English speaking countries and as there have been no similar studies to date that have been carried out in Asian populations the transferability of the intervention beyond western cultures has yet to be ascertained. Therefore, this prospective case series aims to demonstrate proof of concept by exploring the use of a health improvement screening tool (HIP) [19] in Hong Kong. The authors of the HIP state that the measure should be used as a tool for eliciting positive change [19], and therefore this study also aims to seek to establish the potential effects of utilising the HIP by repeating the measure at 12 months follow-up.

### Study aims

•To explore the utility and potential impact of the HIP screening tool in routine clinical practice on the physical health state and health behaviours of people with SMI after 12 months.

## Methods

### Study design

The study utilises a prospective case series study design. The HIP was used as a screening tool at baseline and repeated at 12 months follow-up. In addition to gathering longitudinal data about physical state and health behaviours over one year, the findings were used to inform potential individualised interventions. The following demographic and clinical characteristics were also recorded: age, gender, education, marital status, diagnosis, duration of illness, employment status and prescribed medication.

### Recruitment and selection

The 30 Community Psychiatric Nurses (CPNs) who participated in the study were trained in how to use the HIP and how to conduct the required physical examinations. Each CPN aimed to recruit 5 patients who met the inclusion criteria and complete the HIP with them at baseline and at 12 months follow-up. In line with a consecutive recruitment strategy; CPNs sought informed consent from the patients who they were scheduled to see for routine appointments until a minimum of at least 5 patients agreed for their data to be used.

Patients were approached for consent if they met the following inclusion criteria:

(1) Male or female. Aged from 18–65 years; (2) with a diagnosis of SMI defined by a case-note diagnosis of schizophrenia, schizoaffective disorder, psychotic depression or bipolar affective disorder (type 1 or 2).

Any service user who did not have capacity to provide informed consent was excluded from the study.

### Ethical considerations

Ethical approval was obtained from the Hong Kong New Territories West Cluster Clinical and Research Ethics Committee prior to commencement of the project. Participants who met the inclusion criteria were required to provide written informed consent in order to participate; the participant information sheets reiterated that taking part was entirely voluntary and those patients who declined to take part would not have their clinical treatment negatively influenced.

### Data collection

This study used the HIP [19] as a screening tool. The items and parameters included in the tool were identified using a systematic review of the literature. The gender specific tool contains 27 items which are designed to highlight indicators of physical health risk in people with SMI (Table 1 provides details of the 27 items included on the HIP). Data relating to the psychometric properties of the HIP have not been established/reported; however the face validity, patient acceptability and clinical utility have been demonstrated in a UK population of SMI patients [19,20].

**Table 1:** HIP items

1. Body mass index	15. Breast check (female and male)
2. Waist circumference	16. Menstrual cycle (female)
3. Pulse	17. Smoking status
4. Blood pressure	18. Exercise
5. Temperature	19. Alcohol intake
6. Liver function tests	20. Diet: 5-a-day
7. Lipid levels	21. Diet: fat intake
8. Glucose	22. Fluid intake
9. Cervical smear (women only)	23. Caffeine intake
10. Prostate and testicles check (men only)	24. Cannabis use
11. Sleep	25. Safe sex
12. Teeth	26. Urine
13. Eyes	27. Bowels
14. Feet	28. Sex satisfaction

The parameters for use in Hong Kong required modification in relation to waist circumference and Body Mass Index (BMI) to ensure that they reflected the values recommended for an Asian population. As there has been some debate in the literature about the most relevant BMI cut off points for being defined as overweight in Hong Kong [21], for clarity we present the results of numbers of patients with a BMI of ≥23 kg/m^2^ (the usual cut-off value for being determined as overweight in a Chinese population) and ≥25 kg/m^2^ (the usual cut-off value for being overweight in a European population).

The biometric data (blood tests etc.) required to complete the HIP were obtained from the most recent outpatient records and patients were therefore not required to undergo any investigations that would not normally be considered as part of routine clinical practice. CPNs collected data to ensure that they would be immediately aware of physical health problems that were detected and also be able to recommend health behaviour interventions or refer patients to the appropriate existing clinical service based on the findings from the HIP.

### Data analysis strategy

Data were entered into SPSS version 21. Descriptive statistical analysis was undertaken to establish means/standard deviations and frequencies at baseline and follow-up. Paired samples T-tests were conducted to explore significant differences in the pre and post means of continuous variables, whilst Chi-square or McNemar tests were conducted to determine differences in the categorical variables at baseline and follow-up. Further analysis of variance and binomial logistic regression were conducted to explore the origins of observed change in selected variables.

## Results

**Figure 1:**
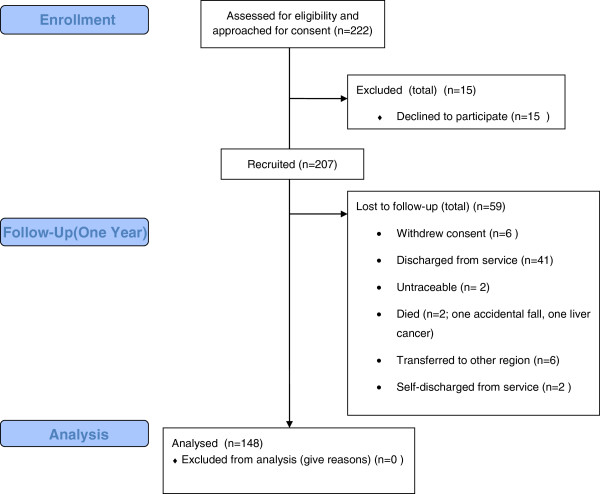
Physical health screening - study flow diagram.

A total of 222 patients were assessed for eligibility and were approached for consent to participate in the study; 15 of these declined to take part and therefore a total of 207 HIPs were completed at baseline during February 2012. The follow-up measures were completed during February and March 2013, for a variety of reasons 59 participants (29%) were lost to follow-up and therefore data from 148 participants were analysed. The specific reasons for attrition are detailed in the study flow diagram (Figure 1).

### Demographic and clinical characteristics of study participants

The demographic and clinical characteristics of participants are presented in Table 2. All patients involved in this study were of Chinese ethnicity and residents of the Hong Kong New Territories. The mean age was 47.14 years (range 21–70; SD 10.61). On average they had been diagnosed with a SMI for 13.36 years (range 0.5-43; SD 10.16); the majority (66%) having a diagnosis of schizophrenia or related disorders and one quarter of participants being diagnosed with a mood-related disorder. Two-thirds of patients were single or divorced, and just under a third (29%) were married. The majority of patients (65%) were educated to secondary school level and above, whilst six had no formal education. Only a quarter of participants were employed with the remainder being unemployed, retired or looking after the family home.

**Table 2:** Demographic and clinical characteristics of participants

**Demographic**	**Number (%)**
**Gender**	
Male	64 (43.2)
Female	84 (56.8)
**Marital status**	
Single	57 (38.5)
Married	44 (29.7)
Widowed	10 (6.8)
Divorced	36 (24.3)
Unknown	1 (0.7)
**Educational level**	
None	6 (4.1)
Primary	45 (30.4)
Secondary	83 (56.1)
University/post graduate	13 (8.8)
Unknown	1 (0.7)
**Diagnosis**	
Schizophrenia	53 (35.8)
Paranoid Schizophrenia	35 (23.6)
Delusional disorder	10 (6.8)
Bipolar affective disorder (BPAD)	15 (10.1)
Psychotic depression	23 (15.5)
Other (i.e. schizoaffective disorder; non-specified psychosis)	10 (6.8)
Unknown	2 (1.4)
**Employment status**	
Unemployed	68 (45.9)
Employed	39 (26.3)
Homemaker	35 (23.6)
Retired/other	5 (3.4)
Unknown	1 (0.7)

### Prescribed medications at baseline and follow-up

Prescribed medications at baseline and follow-up are detailed in Table 3. Prescriptions for psychotropic medications changed very little between both time points. At baseline over 85% of patients in this study were prescribed an antipsychotic medication; the mean number of prescribed antipsychotics was 1.11 (range: 0–3; SD 0.63), the majority of these were atypical antipsychotics and just over 20% were prescribed two or more antipsychotics. Just under a quarter of patients were receiving their antipsychotics as long acting intramuscular injections. At follow-up the mean number of antipsychotic medications prescribed remained the same as at baseline (mean: 1.11; SD 0.65) and just over 10% of patients at both baseline and follow-up were not prescribed any antipsychotics.

**Table 3:** Prescribed Medication at baseline and follow-up

**Medication variable**	**Baseline - number (%)**	**Follow-up - number (%)**	**Significance (P)**
**Number of antipsychotics prescribed**			P = 0.71
0	19 (12.8)	18 (12.2)	
1	95 (64.2)	98 (66.2)	
2	30 (20.3)	29 (19.6)	
3	4 (2.7)	3 (2.0)	
**Prescribed first generation antipsychotics**			P = 0.17
Yes	52 (35.1)	45 (30.4)	
No	96 (64.9)	102 (68.9)	
Unknown		1 (0.7)	
**Prescribed second generation antipsychotics**			P = 0.73
Yes	92 (62.2)	93 (62.8)	
No	56 (37.8)	54 (36.5)	
Unknown		1 (0.7)	
**Prescribed clozapine**			P = 1.00
Yes	13 (8.8)	12 (8.1)	
No	135 (91.2)	136 (91.9)	
**Prescribed long acting antipsychotic injection**			P = 1.00
Yes	34 (23.0)	33 (22.3)	
No	114 (77.0)	114 (77.0)	
Unknown		1 (0.7)	
**Prescribed an antihypertensive**			P = 0.07
Yes	32 (21.6)	21 (14.2)	
No	116 (78.4)	125 (84.5)	
Unknown		2 (1.4)	
**Prescribed a statin**			P = 1.00
Yes	6 (4.1)	5 (3.4)	
No	142 (95.9)	143 (96.6)	
**Prescribed diabetes treatment**			P = 0.04*
Yes	16 (10.8)	8 (5.4)	
No	132 (89.2)	140 (94.6)	

P for significant difference between baseline and follow-up – tested by McNemar test. *Statistically significant at p < 0.05.

The numbers of patients prescribed medications for physical health problems slightly reduced at follow-up; however, McNemar tests reveal that the only statistically significant reduction was in the numbers of people being prescribed treatment for diabetes (p = 0.04). Very few patients were receiving medications for hypercholesterolemia at both time points, whilst prescriptions for hypertension reduced at follow-up from 21% to 14% of patients.

### Cardiovascular risk measurements at baseline and follow-up

Table 4 outlines the mean values of cardiovascular risk measurements at both time points. The mean Body Mass Index (BMI) at baseline and follow-up was just over 25, which suggests being overweight as determined by both European and Asian criteria. Although all the means (except waist circumference and LDL cholesterol) were reduced at follow-up, the paired samples T-tests show that only waist circumference significantly increased at follow-up (t = -2.165; df = 89; p = 0.03). The sparseness of data relating to blood tests that were obtained from outpatients records may account for the lack of statistical significance due to lack of power.

**Table 4:** Mean and SD of cardiovascular risk measurements at baseline and follow-up

	**Baseline mean (SD)**	**Follow-up mean (SD)**	**P-value (two-tailed)**
**BMI** (n = 137)	25.79 (4.72)	25.66 (4.65)	P = 0.55
**Weight** (n = 129)	66.76 (13.63)	66.49 (12.79)	P = 0.67
**Waist circumference - cm** (n = 90)	87.32 (11.84)	89.90 (12.90)	P = 0.03*
**Blood pressure - diastolic** (n = 96)	81.93 (11.30)	80.77 (10.35)	P = 0.40
**Blood pressure - systolic** (n = 96)	127.09 (16.11)	125.05 (14.93)	P = 0.18
**Total cholesterol - mmol/L** (n = 33)	4.64 (1.20)	4.30 (1.42)	P = 0.24
**LDL cholesterol - mmol/L** (n = 33)	2.49 (0.76)	2.80 (1.36)	P = 0.19
**Fasting plasma glucose - mmol/L** (n = 53)	5.99 (2.31)	5.96 (1.76)	P = 0.89
**Triglycerides** (n = 31)	1.49 (0.75)	1.49 (0.77)	P = 0.99

P for significant differences in cardiovascular risk between baseline and follow-up, tested by paired samples T-tests; *Statistically significant at p < 0.05.

### Flagged indicators of cardiovascular risk at baseline and follow-up

Table 5 shows the number of patients’ flagged red or green for a variety of cardiovascular risk indicators. With the exception of waist circumference and raised fasting blood glucose levels the numbers of patients being red-flagged reduced in all other areas at follow-up. Over three quarters were deemed to be overweight in accordance with Asian BMI criterion at baseline and this reduced to just over 71% at follow-up. Although general improvements were observed at follow-up none of the differences are statistically significant at the p < 0.05 level. The deteriorations in waist circumference and blood glucose levels are also not significant, however the McNemar test shows that the increase in numbers of patients with waist circumference measurement suggesting central obesity is approaching significance (p = 0.07).

**Table 5:** Flagged indicators of cardiovascular risk at baseline and follow-up

	**Baseline n (%)**	**Follow-up n (%)**	**P-value**
**BMI overweight - Hong Kong criterion**			P = 0.15
Red flagged (BMI ≥ 23)	113 (76.4)	106 (71.6)	
Green flagged (BMI = 18.50-22.99)	31 (20.9)	40 (27.0)	
Missing	4 (2.7)	2 (1.4)	
**BMI overweight- international criterion**			P = 0.58
Red flagged (BMI ≥ 25)	83 (56.1)	79 (53.4)	
Green flagged (BMI = 18.50-24.99)	65 (43.9)	68 (45.9)	
Missing	0	1 (0.7)	
**Waist circumference**			P = 0.07
Red flagged (males ≥ 90 cm, females ≥ 80 cm)	78 (52.7)	90 (60.8)	
Green flagged (males < 90 cm, females < 80 cm)	68 (45.9)	57 (38.5)	
Missing	2 (1.4)	1 (0.7)	
**Hypertension**			P = 1.00
Red flagged (BP **≥** 140/90)	32 (21.6)	32 (21.6)	
Green flagged (BP < 140/90)	115 (77.7)	116 (78.4)	
Missing	1 (0.7)	0	
**Hypercholesterolemia**			P = 0.38
Red flagged (Total Cholesterol ≥ 6.2 mmol/L)	36 (24.3)	21 (14.2)	
Green flagged (< 6.2 mmol/L)	83 (56.1)	83 (56.1)	
Missing	29 (19.6)	44 (29.7)	
**Raised LDL cholesterol**			P = 0.21
Red flagged (LDL ≥ 4.1 mmol/L)	29 (19.6)	13 (8.8)	
Green flagged (< 4.1 mmol/L)	95 (64.2)	93 (62.8)	
Missing	24 (16.2)	42 (28.4)	
**Raised fasting plasma glucose**			P = 0.21
Red flagged (Glucose - ≥ 7.00 mmol/L)	16 (10.8)	21 (14.2)	
Green flagged (< 7.0 mmol/L)	116 (78.4)	83 (56.1)	
Missing	16 (10.8)	44 (29.7)	
**Raised triglycerides**			P = 1.00
Red flagged (Triglycerides - ≥ 2.2 mmol/L)	39 (26.4)	24 (16.2)	
Green flagged (< 2.2 mmol/L)	84 (56.8)	75 (50.7)	
Missing	25 (16.9)	49 (33.1)	

P for significant differences in cardiovascular risk between baseline and follow-up, tested by McNemar test.

### Patient’s health behaviours

Table 6 outlines the patients’ health behaviours at both baseline and follow-up. Similarly to the cardiovascular risk factors results, all health behaviours (except sleep disruption) had improved at follow-up. However, the only statistically significant result relates to an improvement in the numbers of patients at follow-up reporting that they exercise at recommended levels (p = 0.02). The numbers of patients reporting sleep disruption increased from 27% to 34% at follow-up, however this is not significant at the p < 0.05 level (p = 0.15).

**Table 6:** Health behaviours of participants at baseline and follow-up

**Health behaviour**	**Baseline n (%)**	**Follow-up n (%)**	**P-value**
**Alcohol intake**			P = 0.79
Red flagged (≥ 4 units daily)	9 (6.1)	7 (4.7)	
Green flagged (< 2-3 units/day)	128 (86.5)	141 (95.3)	
Unknown	11 (7.4)	0	
**Smoking status**			P = 0.45
Red flagged (smoker/passive smoker)	40 (27.0)	36 (24.3)	
Green flagged (non-smoker/non passive smoker)	108 (73.0)	112 (75.7)	
**Caffeine intake**			P = 0.79
Red flagged (≥ 600 mg/day)	8 (5.4)	6 (4.1)	
Green flagged (< 600 mg/day)	130 (87.8)	142 (95.9)	
Unknown	10 (6.8)	0	
**Exercise**			P = 0.02*
Red flagged (< 30 minutes a day for 5 days per week)	95 (64.2)	78 (52.7)	
Green flagged (≥ 30 minutes a day for 5 days per week)	50 (33.8)	69 (46.6)	
Unknown	3 (2.0)	1 (0.7)	
**Diet fruit and vegetables**			P = 0.08
Red flagged (≤ 2 daily portions)	41 (27.7)	31 (20.9)	
Green flagged (≥ 5 daily portions)	106 (71.6)	117 (79.1)	
Unknown	1 (0.7)		
**Diet Fats**			P = 0.74
Red flagged (≥ 2 portions per day)	30 (20.3)	28 (18.9)	
Green flagged (≤ 1 portion per day)	114 (77.0)	120 (81.1)	
Unknown	4 (2.7)	0	
**Disrupted sleep**			P = 0.15
Red flagged (>8 h or <3 h)	40 (27.0)	51 (34.5)	
Green flagged (7 – 8 hours)	107 (72.3)	96 (64.9)	
Unknown	1 (0.7)	1 (0.7)	

P for significant differences between health behaviours at baseline and follow-up, tested by McNemar test; * = Statistically significant at p < 0.05.

### Secondary analyses

In order to further explore the origins of the significant change we observed in waist circumference, we conducted analyses of variance and binomial logistic regression. In the first instance, we used five classifications of BMI values that suggest different levels of obesity (underweight ≤ 18.49 kg/m^2^, normal weight = 18.50-24.99 kg/m^2^, overweight = 25-29.99 kg/m^2^, obese = 30-39.99 kg/m^2^, morbid obesity ≥ 40 kg/m^2^) as a between subjects factor to detect mean differences in waist circumference change (computed as the difference between measurements at baseline and follow-up). The ANOVA showed no significant difference between the five BMI categories in terms of waist change F (4,85) = 0.611, p = 0.66. We then then conducted a binomial logistic regression in order to predict levels of waist change (where no changes or decreases in waist circumference measurements between baseline and follow up were recoded as 0 and increases were recoded as 1) from a mixture of categorical (gender, diagnosis, occupation, prescribed medication) and continuous (age, duration of illness) predictors. None of the models calculated (simultaneous or stepwise) resulted in a fitting model able to correctly predict cases much beyond chance.

## Discussion

The physical health state of the patients involved in this study is markedly worse than the general Hong Kong population. Although direct comparisons are complicated by differing settings and age distributions, data reported by Ko et al., [21] suggests that 44% of women and 68% of men aged 40–50 years in the Hong Kong working population were overweight (defined as a BMI ≥23) and the current study indicates that at baseline 77% of men and 76% women are overweight. In terms of waist circumference; Ko et al., [22] estimate the prevalence of central obesity as 30% for men and 29% for women, whilst our study shows that 42% of women and 61% of men were centrally obese at baseline. Therefore, these results indicate that men are more than twice as likely to have central obesity and women are over 1½ times more likely to be overweight compared to the general population; findings which are supported by a number of other studies conducted in different countries that estimate the relative risk of obesity in people with schizophrenia as being between 1.5 and 2 [6,23,24].

This study shows that the challenges mental health practitioners face when promoting physical health in Hong Kong are comparable to those observed in western countries. The prevalence of being overweight in this study is broadly similar to that of SMI patients screened using the HIP in Scotland; Shuel et al., [20] reported that 77% of patients were overweight (72% were overweight at follow-up in the present study). Whilst central obesity was slightly less prevalent in this study (61% compared to 70% in Scotland), rates of hypertension were higher (21% verses 13%) and raised plasma glucose levels were also more common (14% compared to 10% in Scotland). Interestingly, with the exception of alcohol use and fruit/vegetable consumption the self-reported health behaviours of the patients in this study are somewhat worse than those reported by Shuel et al., [20]; most noteworthy is the 64% of patients in Hong Kong who at baseline were not exercising at recommended levels (in comparison with 29% in Scotland).

Our results show that after one year there were some encouraging improvements in relation to both health behaviours and indicators of cardiovascular risk. Although not statistically significant, most areas of cardiovascular risk improved over the course of the study. Disappointingly, despite some slight improvement in BMI, the mean waist circumference measurement of participants significantly increased by 2.58 cm at follow-up (p = 0.03). This result may be confounded by the lack of objective verification that all the waist circumference measurements conducted by clinicians adhered to the suggested protocol. Although the clinicians carrying out measurements were instructed to place the tape measure across the umbilicus, there are at least 6 established alternative waist measurement points that are used routinely in practice, which are equally predictive of cardiovascular risk but which will all produce different results [25].

In terms of the health behaviours which are modifiable physical health risk factors; all areas showed minor improvements, including a 7% increase in the number of patients eating sufficient fruit and vegetables, but only exercise improved to a statistically significant level (p = 0.02). Therefore, despite some positive outcomes in health behaviours, with the exception of waist circumference the physical state of patients remained largely unchanged over the 12 month period of the study. Our findings are similar to some of the previous exercise and nutritional studies which have shown that although health behaviours improved in patients with SMI, this did not manifest in significant reductions in BMI, waist circumference or body weight, however the interventions may have prevented further deterioration [26-29].

Given the poor physical state of patients at commencement of the study it would be appropriate to assume that physical health would have deteriorated further after 1 year and this could indicate that the enhanced screening using the HIP prevented some further decline. This hypothesis is supported by the observed marked physical health deterioration in treatment as usual (TAU) SMI control groups in some previous studies, for example: Littrell et al., [30] report that the mean weight in the TAU group steadily increased over 16 weeks in a psycho-educational study; the TAU group in a healthy living programme showed a 8.1% gain in BMI over one year [31] and mean waist circumference increased by 1.2 cm over three months in the TAU group in a “solution for wellness” group intervention programme conducted by Porsdal et al., [32].

Our results highlight that there was little change in the numbers and types of psychotropic medications prescribed over the duration of the study. But there was a significant reduction in the numbers of patients being treated with medications for diabetes; this is a promising finding and could suggest that the improvements in exercise and diet have resulted in better glycaemic control, necessitating a reduced need for medication. However, due to the lack of control group, abundance of extraneous variables and in the absence of objective measures of health behaviour changes, it is not possible to confidently attribute improvements in the need for diabetes treatments to the enhanced screening programme. Despite the methodological shortfalls of the present study, our hypothesis may be supported by many previous research studies which show that people with sedentary lifestyles and poor dietary habits have a substantially increased risk of diabetes [33,34], and also that exercise and diet interventions can reduce the risk of diabetes more dramatically than the effects of medication [35].

The study demonstrates that use of the HIP as a screening tool in Hong Kong is both feasible and useful to identify areas where physical health requires intervention. As a potential secondary gain enhanced physical health monitoring may also address concerns about treatment; Bressington et al., [36] demonstrated that patients and families in Hong Kong are concerned about the effects of psychotropic medications on patients’ physical health, if there is increased attention in this area perhaps this will go some way to reinforcing that these concerns are being taken seriously and hence provide some reassurance in this regard. The numbers of patients who agreed to participate and completed the study suggest that an increased clinical focus on physical health problems is acceptable from a patient perspective, but in order to maximise the potential of health behaviour interventions clinicians need to ensure that there are adequate services available to provide ongoing support; which will entail a co-ordinated interprofessional intensive effort from health care workers.

### Strengths and limitations of the study

We are unable to make assertions from our results about causality due to the prospective case series design of the study. Due to the lack of a control group and absence of objective measures of behaviour it is not possible to confidently ascribe the improvements in health behaviours or lack of deterioration in physical health to the HIP screening programme; the changes in physical state may result from natural fluctuations, response to prescribed medical treatments or other influences. Patients’ self–reported their health behaviours, and therefore potentially an overly positive picture may have been presented in an effort to please their keyworkers. The recruitment process may also have introduced selection bias and the sparseness of data relating to blood tests may have resulted in lack of power and therefore statistical significance may be affected.

Despite the methodological limitations of this study, the results demonstrate that enhanced physical health screening using the HIP is feasible and acceptable in this setting; if at the very least such screening programmes prevent deterioration in physical health then this would be a clinically meaningful outcome. In terms of future research, this study shows that it is possible to recruit and retain patients with SMI in health screening studies, and as clinicians were able to use the HIP to integrate relatively inexpensive health screening into routine clinical practice it would be sensible conduct further studies which utilise a randomised controlled trial design and employ a randomised recruitment strategy.

## Conclusions

Annual health checks using the HIP are useful in monitoring the physical health of patients with SMI and are also effective in identifying individualised targets for clinical intervention. The results show that over the 12-month duration of the study the amounts of self-reported exercise improved and the numbers of patients prescribed diabetes medications reduced, but mean waist circumference increased. In this instance the enhanced screening programme does not seem to have significantly improved patients’ cardiovascular risk factors, but may have resulted in the prevention of further health deterioration.

## Competing interests

RG has received honoraria and provided consultancy to AstraZeneca, Bristol-Myers Squibb, Jannsen Cilag, Eli Lilly and Co., Otsuka Pharmceutical Europe Ltd, Pfizer; received honoraria from AstraZeneca, Bristol-Myers Squibb, Jannsen-Cilag, Eli Lilly and Co., Otsuka Pharmaceutical Europe Ltd, Lundbeck, Pfizer, Wyeth and had research funding from AstraZeneca, the Medical Research Council, the National Institute for Mental Health, the Department of Health and Comic Relief. DB has received honorarium payments for educational consultancy from Lundbeck and Jannsen-Cilag. The other authors declare no potential conflicts of interest.

## Authors’ contributions

DB designed the study, contributed towards data input, jointly analysed data, interpreted the data analysis and was lead for the writing of the article. JM was data collection lead, contributed towards the study design and commented on the final paper. RG provided advice on study design, delivered the training, advised on data analysis and contributed towards the final paper. EC provided advice on study design, advised on data analysis/interpretation and contributed towards the final paper. SB jointly input data, helped interpret the data analysis and contributed to the final paper. SH provided statistical advice on the data analysis strategy, jointly analysed data and contributed to the final paper. All authors read and approved the final manuscript.

## Pre-publication history

The pre-publication history for this paper can be accessed here:

http://www.biomedcentral.com/1471-244X/14/57/prepub
